# Tannic acid prevents post-weaning diarrhea by improving intestinal barrier integrity and function in weaned piglets

**DOI:** 10.1186/s40104-020-00496-5

**Published:** 2020-09-01

**Authors:** Jie Yu, Yanyan Song, Bing Yu, Jun He, Ping Zheng, Xiangbing Mao, Zhiqing Huang, Yuheng Luo, Junqiu Luo, Hui Yan, Quyuan Wang, Huifen Wang, Daiwen Chen

**Affiliations:** 1 Animal Nutrition Institute, Sichuan Agricultural University, Chengdu, 611130 People’s Republic of China; 2 Key Laboratory for Animal Disease-Resistance Nutrition, Ministry of Education of China, Chengdu, 611130 People’s Republic of China

**Keywords:** Intestine barrier, Post-weaning diarrhea, Tannic acid, Weaned piglets

## Abstract

**Background:**

Tannic acid (TA) is potential to reduce diarrhea in weaning pigs, but knowledge about the influence of TA on intestinal barrier integrity and function is still scarce. This experiment was conducted to investigate the effects of dietary TA supplementation on growth performance, diarrhea rate, intestinal barrier integrity and function of weaned pigs.

**Methods:**

A total of 108 crossbred (Duroc × Landrace × Yorkshire) piglets, with an initial average body weight of 6.60 ± 0.27 kg, were allotted to 3 groups (6 pigs/pen and 6 replicates/group) in a randomized complete block design according to their gender and body weight. Piglets were fed the basal diet with 0 (control, CON), 0.2% and 1.0% TA, respectively. The trial lasted for 28 d.

**Results:**

Compared with the CON group, dietary 0.2% and 1.0% TA supplementation didn’t affect ADFI, ADG and F:G (*P* > 0.05), but reduced diarrhea rate, diarrhea index and diarrhea score of piglets (*P* < 0.05), reduced diamine oxidase (DAO) activity and D-lactic acid concentration in serum (*P* < 0.01). The higher occludin expression and localization were observed in the duodenum, jejunum and ileum after supplementation with 0.2% or 1.0% TA (*P* < 0.05). Adding 0.2% TA to diet significantly decreased crypt depth, increased villus height/crypt depth ratio in the duodenum (*P* < 0.05), and dietary 1.0% TA tended to decrease crypt depth (*P* < 0.10) and significantly decreased villus height (*P* < 0.05) of the ileum. Moreover, lower malondialdehyde content in the ileum was detected in the pigs fed 1.0% TA (*P* < 0.05). In the duodenum, both 0.2% and 1.0% TA groups had higher occludin (*OCLN*) mRNA and 0.2% TA group had higher zonula occludens-2 (*ZO-2*) level (*P* < 0.05). Meanwhile, dietary 1.0% TA supplementation tended to up-regulate *OCLN* mRNA levels in the jejunum (*P* < 0.10) and 0.2% TA supplementation tended to up-regulate zonula occludens-1 (*ZO-1*) mRNA levels in the ileum (*P* < 0.10).

**Conclusion:**

In conclusion, dietary supplementation of 0.2% or 1.0% TA could effectively alleviate post-weaning diarrhea without altering growth performance in weaned piglets, which might be achieved by improving intestinal barrier integrity and function.

## Background

Post-weaning diarrhea usually results in high morbidity and mortality of piglets, which is a serious issue in pig production. The subsequent decline in growth rate has brought enormous economic losses in swine industry. Diarrhea in weaning piglet is mainly caused by weaning stress, lactogenic immunity deprivation, diet and environmental changes. Stress associated with early weaning leads to continuous impairment of intestinal barrier function in pigs [1]. The intestinal barrier consists of a single layer of columnar epithelium and intercellular tight junctions of enterocytes [2], which serves as an important defense mechanism against hostile environment in the intestinal lumen. The complete intestinal barrier not only allows passage of select solutes that are beneficial to the host, but also effectively prevents the passage of antigens, bacterial toxins, and pathogens [3]. Damage in the intestinal barrier, characterized by increased intestinal permeability, usually augments the risk of enteric infection, promotes the translocation of luminal bacteria, toxins, and antigens into subepithelial tissues, and finally results in inflammatory reactions and gastrointestinal diseases [2]. These are the most common pathogenic factors of severe diarrhea in piglets. Therefore, the integrity and function of intestinal barrier play an important role in alleviating diarrhea of weaned piglets.

For the sake of animal food safety and environmental protection, the era of banning antibiotics has come and the policy of limiting high zinc and high copper has been implemented. Therefore, it is extremely urgent to seek effective and natural pollution-free alternatives like plant extracts as substitutes for antibiotics, zinc oxide, high copper, etc. Tannic acid (TA) is one of the typical representatives of high molecular weight polyphenol compounds that mainly exist in a wide variety of plants, such as plant-derived feeds, grains, and fruits [4]. TA was usually considered as an anti-nutritional factor (ANF) in the past because it forms complexes with proteins, polysaccharides, digestive enzymes and metal ions, which are not conducive to the digestion and absorption of nutrients by animals and even considered to be toxic [5, 6]. Interestingly, some recent studies have shown that dietary TA has no effect or has some beneficial effect on the nutrient digestibility and growth performance of animals, which is contrary to the previous public cognition of TA [7, 8]. In addition, many medicinal plants with effect on mitigating diarrhea have revealed the presence of TA, like Gallnut contains massive TA, which even can reach 50–70% of its weight [9]. Subsequently, the anti-diarrhea, anti-oxidation, microbial homeostasis regulation and other effects of TA have been found one after another, which have attracted extensive attention of animal nutritionists [4, 10].

TA has a strong convergence effect in the gastrointestinal tract of pigs and poultry, which slows down intestinal peristalsis, strengthens colonic water and electrolyte reabsorption, and further alleviates the occurrence of diarrhea [9, 11]. Moreover, TA has been proved to improve intestinal damage, increase villus height [12] and reduce crypt depth [13], thus improving the integrity of intestinal morphology and structure. However, knowledge about the influence of dietary supplementation of TA in piglets on intestinal barrier integrity and function is still scarce. Therefore, this study was aimed to investigate the effects of dietary TA supplementation on growth performance, diarrhea rate, intestinal barrier integrity and function of weaned piglets. In this way, we try to explain the effects and the underlying mechanism of TA for alleviating post-weaning diarrhea.

## Methods

The experimental procedures used in this study were approved by the Institutional Animal Care and Use Committee of Sichuan Agricultural University.

### Experimental animals, diet and design

A total of 108 crossbred piglets (Duroc × Landrace × Yorkshire, weaned at 21 ± 1 d of age), with an initial average body weight of 6.6 ± 0.27 kg, were allocated to 3 groups (6 pigs/pen and 6 replicates/group) on the basis of their gender and body weights. Piglets were fed the basal diet supplemented with 0 (control, CON), 0.2% and 1.0% TA, respectively. These TA levels were obtained by adding tannalbin containing 51% TA and 40.17% protein. Namely, tannalbin was added to the diet for 0, 0.4% and 2.0% at the expense of the soybean meals in the equal amount. Tannalbin, a compound of tannic acid with protein was provided by Guangzhou Insighter Biotechnology Co., Ltd. (GuangZhou, China) and in which tannic acid is extracted from Gallnut. According to pharmacopoeia, tannalbin decomposes into TA after entering the small intestine. The feeding experiment lasted for 28 d. The basal diet was formulated according to the National Research Council (NRC 2012) recommendations to meet or exceed the nutritional requirements (Table 1). The analyzed levels of TA in experimental diets for 0.2% and 1.0% TA groups were 1973.09 mg/kg, 12,004.84 mg/kg, respectively. All pigs had free access to feed and water in this 28-d experimental period. The ambient temperature was maintained at 26 ± 2 °C, and relative humidity was controlled at 60% ± 5%.

**Table 1:** Ingredients composition and nutrient levels of basal diets (as-fed basis)

Item	%	Calculated composition^a^	%
Corn	31.37	DE, MJ/kg	14.69
Extruded corn	29.15	CP	18.57
Soybean meal	8.00	Ca	0.74
Fermented soybean meal	5.00	Total P	0.59
Extruded soybean	4.00	Available P	0.42
Soybean protein concentrate	5.00	*D*-Lysine	1.31
Soybean oil	1.50	*D*-Methionine	0.44
Sucrose	3.00	*D*-Methionine + Cystine	0.69
Whey powder	6.70	*D*-Threonine	0.79
Fish meal	3.50	*D*-Tryptophan	0.22
Salt	0.40		
*L*-Lysine HCl	0.42		
*DL*-Methionine	0.15		
*L*-Threonine	0.11		
Tryptophan	0.02		
Choline chloride	0.10		
Limestone	0.75		
Dicalcium phosphate	0.58		
Vitamin premix^b^	0.05		
Mineral premix^c^	0.20		

^a^ Values are calculated

^b^ The premix provides following per kilogram of diet: vitamin A, 15,000 IU; vitamin D_3_, 5,000 IU; vitamin E, 40 mg; vitamin K, 5 mg; vitamin B_1_, 5 mg; vitamin B_2_, 12.5 mg; vitamin B_6_, 6 mg; vitamin B_12_, 0.06 mg; folic acid, 2.5 mg; nicotinic acid, 50 mg; *D*-pantothenic acid, 25 mg; *D*-biotin, 0.25 mg

^c^ The premix provides following per kilogram of diet: Fe (as ferrous sulfate), 100 mg; Cu (as copper sulfate), 6 mg; Mn (as manganese sulfate), 4 mg; Zn (zinc sulfate), 100 mg; I (potassium iodide), 0.14 mg; Se (as sodium selenite), 0.35 mg

### Growth performance and diarrhea

All piglets were individually weighed at the beginning (0 d), middle (14 d) and end (28 d) of the experiment after 12 h of fasting, and average daily gain (ADG) was calculated. The feed intake of each pen was recorded every day to calculate the average daily feed intake (ADFI), and feed to gain ratio (F:G) was calculated using feed intake and body weight gain. The incidence of diarrhea for each pen was observed and recorded at 09:00 and 20:00 h each day during the experimental period. The incidence and severity of piglet diarrhea were assessed by scoring fecal consistency: Scores were 0 = normal, firm feces; 1 = soft feces, possible slight diarrhea; 2 = definitely unformed, moderately fluid feces; 3 = very watery and frothy diarrhea. Piglets were considered to be diarrheic when the diarrhea score was 2 or above, and data were reported as a cumulative score for all pigs on each day [14, 15]. Diarrhea rate was calculated according to the formula [16]: diarrhea rate (%) = Σ (the number of pigs with diarrhea per pen × days of diarrhea)/(total number of piglets × 28 d) × 100. The diarrhea index was calculated in accordance to our previous report [17]: diarrhea index = sum of diarrhea scores of pigs per pen/(number of piglets per pen × total days).

### Sample collection

In the morning of the 29^th^ day after fasting for 12 h, six pigs from each group (one pig per pen) were selected based on average body weight. Blood samples were collected from anterior cava vein into vacuum tubes without anticoagulant, and were then centrifuged at 3,500×*g* for 10 min at 4 °C. The serum was separated into centrifuge tubes and stored at − 20 °C for further analysis. After the blood collection was completed, the same 18 pigs were euthanized with intravenous injection of chlorpromazine hydrochloride (3 mg/kg body weight) reported by Chen et al. [18]. The abdominal cavity of the piglets was then opened, and duodenum, jejunum and ileum were quickly separated on the basis of anatomical structures. Intestinal tissue segments of about 2 cm were immediately separated from the proximal sections of the duodenum, jejunum and ileum with carefulness to avoid squeezing, and fixed in 4% paraformaldehyde solution for intestinal morphology and immunofluorescence analysis. The remaining duodenum and ileum segments and the jejunum segment of about 10 cm (each pig was selected at the same site) were cut longitudinally, and gently washed by 0.9% of pre-cooled saline. Then, the intestinal mucosa was gently scraped by a sterile microscope glass slide into a sterile frozen storage tube (a new glass slide was required for each intestinal segment and the whole process was operated on ice), and then stored to − 80 °C so as to facilitate the determination of gene expression and MDA content.

### Serum parameter measurements

The *D*-lactic acid concentration and diamine oxidase (DAO) activity in serum were measured spectrophotometrically by using the corresponding ELISA kits (Jiangsu Meimian Industrial, Inc., Jiangsu, China) according to the manufacturer’s instructions.

### MDA content in serum and intestinal mucosa

The MDA content in serum was directly detected by using the commercial reagent kits (Nanjing Jiancheng Bioengineering Institute, Jiangsu, China) with UV-VIS Spectrophotometer (UV1100, MAPADA, Shanghai, China) according to the manufacturer’s instructions. The frozen small intestinal mucosal samples were weighed (approximately 0.5 g) and homogenized in pre-cooled physiological saline solution (1:9, weight/volume). The supernatant solution was collected into centrifuge tubes after homogenate mixture was centrifuged at 3,500×*g* for 10 min at 4 °C to determine intestinal MDA content. MDA content in small intestinal mucosa was detected using commercial kits (Nanjing Jiancheng Bioengineering Institute, Jiangsu, China) with a UV-VIS Spectrophotometer (UV1100, MAPADA, Shanghai, China) according to the manufacturer’s instructions. Total protein concentration of supernatant solution was determined as the protein standard using the Braford brilliant blue method by the commercial kits (Nanjing Jiancheng Bioengineering Institute, Jiangsu, China). All samples were measured in triplicate.

### Intestinal morphology

The morphology including villus height, crypt depth, intestinal wall thickness and mucosal thickness of duodenum, jejunum and ileum were measured after samples were fixed in 4% paraformaldehyde solution for 24 h according to the procedure described in the study of He et al. [17]. Simply speaking, the fixed intestinal samples were dehydrated in ethanol, cleared in xylene, and embedded in paraffin wax. Then, the samples were transverse sectioned at a 5-μm thickness and installed on glass slides. Paraffin sections were dewaxed to water with xylene, ethanol and distilled water, and stained with hematoxylin and easin. Finally, the slices were sealed with neutral gum after being dehydrated again for a light microscopy examination. A minimum of 10 well-oriented, intact villi and crypt was measured with Image-pro plus 6.0 (Media Cybernetics, Inc., Rockville, MD, USA) for each intestinal sample. In the same way, the thickness of mucosal layer and intestinal wall was measured at 10 sites of each sample.

### Immunofluorescence analysis

Immunofluorescence assay was used to determine the localization and expression of occludin in duodenal, jejunal and ileal tissue. The preparation steps of paraffin sections were the same as intestinal morphology analysis. After dewaxed to water with xylene, ethanol and distilled water, the tissue sections were placed in ethylene diamine tetraacetic acid (EDTA) buffer (pH 8.0, Servicebio Technology Co., Ltd., Wuhan, China) for antigen retrieval. And then, the slides were washed 3 times in phosphate buffer saline (PBS, pH 7.4). A histochemical pen (Gene Technology Co., Ltd., Shanghai, China) was used to draw a circle around the tissue to prevent the antibody from flowing away, autofluo quencher were added for 5 min, and then incubated with 3% BSA. Subsequently, the tissue sections were incubated overnight at 4 °C with rabbit anti-occludin polyclonal antibody (1:200; Abcam plc., Cambridge, UK). After slides were washed 3 times with PBS, the goat anti-rabbit IgG-Cy3 secondary antibody (Servicebio Technology Co., Ltd., Wuhan, China) was added to the circle to cover the tissues and then incubated at room temperature in the dark for 50 min. Slides were washed with PBS and the 4′,6-diamidino-2-phenylindole (DAPI, Servicebio Technology Co., Ltd., Wuhan, China) was dripped into the circle and incubated at room temperature in the dark for 10 min. Finally, slides were washed with PBS and sealed with anti-fluorescence quenching sealer (Servicebio Technology Co., Ltd., Wuhan, China). All slides were observed, and the images were collected using a confocal scanning microscope (NIKON ECLIPSE TI) and NIKON DS-U3 software. The localization and expression of occludin in the small intestine was analyzed by fluorescence evaluation. DAPI stain in the images indicates live cells.

### Relative quantitative real-time PCR

The frozen small intestinal mucosa sample (approximately 0.1 to 0.2 g) was ground into powder (liquid nitrogen was continuously added during grinding to maintain a lower temperature) and added into a sterile centrifuge tube containing 1 mL RNAiso Plus reagent (TaKaRa, Dalian, China). Then, the total RNA was extracted from duodenal, jejunal and ileal mucosa following the manufacturer’s instructions. For each sample, the concentration and quality of total RNA were verified by using a spectrophotometer (NanoDrop Technologies, Inc., Wilmington, DE, USA) at 260 and 280 nm. The optical density (OD) ratio (260 nm/280 nm) was between 1.8 and 2.0. Moreover, the integrity of RNA was determined by formaldehyde agarose gel electrophoresis. RNA of each sample was reverse-transcribed using the PrimeScript™ RT reagent Kit (TaKaRa Biotechnology Inc., Dalian, China) according to the manufacturer’s directions.

Quantitative real-time PCR (qRT-PCR) was performed to determine the mRNA expression levels of zonula occludens-1 (*ZO-1*), zonula occludens-2 (*ZO-2*), occludin (*OCLN*), claudin-1 (*CLDN-1*) and claudin-2 (*CLDN-2*) using an CFX96™ Real-Time PCR Detection System (Bio-Rad Laboratories, Inc., Hercules, CA) and SYBR Green reagents (TakaRa, Dalian, China) according to the manufacturer’s protocol. The specific primers for them were commercially synthesized and purchased from Sangon Biotech Co., Ltd. (Shanghai, China) and were listed in Table 2. The 10 μL qRT-PCR system consisted of 5 μL SYBR Green (TaKaRa, Dalian, China), 0.5 μL forward primer, 0.5 μL reverse primer, 3 μL nuclease-free H_2_O and 1 μL cDNA template. The reactions were performed at 95 °C for 30 s, 40 cycles of denaturization at 95 °C for 5 s, and annealing at annealing temperature for 30 s with a final extension at 72 °C for 5 min. And *GAPDH* was used as the reference gene transcript. The specificity of PCR amplification was confirmed by melting curve analysis. All samples were repeated in triplicate and results of the relative expression ratio of target genes relative to the reference gene were calculated using the 2^–ΔΔCt^ method [19].

**Table 2:** Primers used for real-time quantitative PCR

Gene	Accession No.	Primer sequences (5′→3′)	Size, bp
*ZO-1*	XM_005659811.1	F: CAGCCCCCGTACATGGAGA	114
		R: GCGCAGACGGTGTTCATAGTT	
*ZO-2*	NM_001206404.1	F: ATTCGGACCCATAGCAGACATAG	90
		R: GCGTCTCTTGGTTCTGTTTTAGC	
*OCLN*	NM_001163647.2	F: CTACTCGTCCAACGGGAAAG	158
		R: ACGCCTCCAAGTTACCACTG	
*CLDN-1*	NM_001258386.1	F: GCCACAGCAAGGTATGGTAAC	140
		R: AGTAGGGCACCTCCCAGAAG	
*CLDN-2*	NM_001161638.1	F: GCATCATTTCCTCCCTGTT	156
		R: TCTTGGCTTTGGGTGGTT	
*GAPDH*	NM_001206359.1	F: TGAAGGTCGGAGTGAACGGAT	114
		R: CACTTTGCCAGAGTTAAAAGCA	

*ZO-1* Zonula occluden 1, *ZO-2* Zonula occluden 2, *OCLN* Occludin, *CLDN-1* Claudin 1, *CLDN-2* Claudin 2, *GAPDH* Glyceraldehyde-3-phosphate dehydrogenase, *F* Forward, *R* Reverse

### Statistical analysis

Data were analyzed by a one-way ANOVA analysis using the GLM procedure of SAS 9.2 (SAS Institute Inc., Cary, NC, USA) in a completely randomized design. Each pen formed the experimental unit for pig growth performance and diarrhea indicators, and the selected pig served as the experimental unit for other traits. All data for diarrhea evaluation were converted by arcsine square root transformation for statistics. Results were presented as means and standard error of means (SEM). A Duncan’s multiple comparison was applied to analyze the differences among groups. Linear and Quadratic contrasts were used to determine the dose effect of tannic acid in weaned piglets. A *P*-value of *P* < 0.05 was considered statistically significant, and 0.05 < *P* ≤ 0.10 were accepted as representing tendencies to differences.

## Results

### Growth performance and diarrhea

As shown in Table 3, there were no differences in ADFI, ADG and F:G between the 3 groups of pigs (*P* > 0.05). As shown in Table 4, the addition of 0.2 and 1.0% TA to the diet linearly reduced the diarrhea rate, diarrhea index and diarrhea score of weaned piglets compared to the CON group (*P* < 0.05).

**Table 3:** Effects of tannic acid (TA) on growth performance in weaned piglets

Item	Added tannic acid, %			SEM	*P*-value		
	0	0.2	1.0		ANOVA	Linear	Quadratic
Initial BW, kg	6.6	6.6	6.6	0.27	1.000	0.996	0.999
Final BW, kg	12.7	13.4	12.7	0.69	0.727	0.758	0.469
0 to 14 d							
ADFI, g	265.6	281.8	271.9	24.44	0.895	0.976	0.644
ADG, g	154.3	165.1	151.6	16.20	0.826	0.760	0.598
F:G ratio	1.76	1.71	1.80	0.06	0.587	0.442	0.487
15 to 28 d							
ADFI, g	528.7	560.6	540.2	24.86	0.661	0.975	0.372
ADG, g	281.8	318.4	280.8	20.58	0.364	0.614	0.188
F:G ratio	1.88	1.79	1.94	0.07	0.294	0.264	0.271
0 to 28 d							
ADFI, g	397.1	421.2	406.1	23.08	0.761	0.974	0.467
ADG, g	218.1	241.7	216.2	16.44	0.490	0.641	0.277
F:G ratio	1.83	1.76	1.89	0.05	0.152	0.148	0.171

CON, piglets receiving a basal diet; CON + 0.2% TA, piglets receiving a basal diet supplemented with 0.2% TA; CON + 1.0% TA, piglets receiving a basal diet supplemented with 1.0% TA; ADFI, average daily feed intake; ADG, average daily gain; F:G, feed:gain ratio

**Table 4:** Effects of tannic acid (TA) on diarrhea rate, diarrhea index and diarrhea score in weaned piglets

Item	Added tannic acid, %			SEM	*P*-value		
	0	0.2	1.0		ANOVA	Linear	Quadratic
0 to 14 d							
Diarrhea rate, %	19.4^a^	13.0^ab^	8.7^b^	2.46	0.035	0.013	0.413
Diarrhea index	0.5	0.3	0.2	0.07	0.076	0.034	0.335
Diarrhea score	14	11	8	0.42	0.061	0.024	0.409
15 to 28 d							
Diarrhea rate, %	16.8^a^	10.2^a^	2.9^b^	1.99	0.0004	0.0001	0.392
Diarrhea index	0.4^a^	0.2^a^	0.1^b^	0.05	0.001	0.0003	0.516
Diarrhea score	11^a^	9^a^	3^b^	0.28	0.001	0.0002	0.628
0 to 28 d							
Diarrhea rate, %	18.1^a^	11.6^a^	5.8^b^	2.05	0.004	0.001	0.353
Diarrhea index	0.4^a^	0.3^ab^	0.1^b^	0.05	0.009	0.003	0.354
Diarrhea score	13^a^	10^ab^	5^b^	0.31	0.008	0.003	0.452

CON, piglets receiving a basal diet; CON + 0.2% TA, piglets receiving a basal diet supplemented with 0.2% TA; CON + 1.0% TA, piglets receiving a basal diet supplemented with 1.0% TA

^a,b^Mean values with unlike superscript letters were significantly different (*P* < 0.05)

### Intestinal permeability and the localization of occludin

**Fig. 1:**
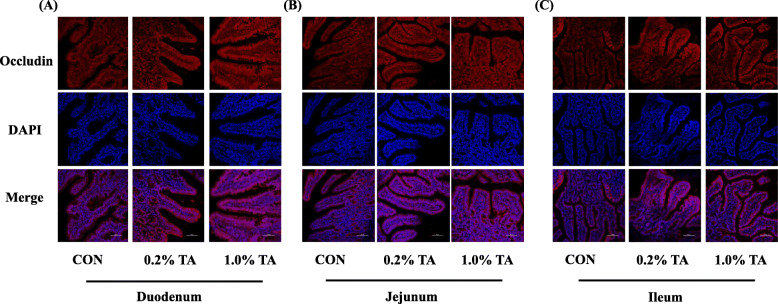
Effect of tannic acid (TA) on expression and localization of occludin protein in small intestine of weaned piglets (scale bar: 100 μm). The localization of tight junction protein occludin in duodenum (**a**), jejunum (**b**) and ileum (**c**) of weaned piglets was visualized using immunofluorescence technique. The localization of occludin (red), DAPI (blue), as well as merged occludin and DAPI are shown. DAPI stain indicates live cells. CON, piglets receiving a basal diet; CON + 0.2% TA, piglets receiving a basal diet supplemented with 0.2% TA; CON + 1.0% TA, piglets receiving a basal diet supplemented with 1.0% TA

The effects of dietary TA supplementation on serum parameters are presented in Table 5. Compared with the CON group, 0.2% and 1.0% TA in the diet significantly reduced DAO activity and D-lactic acid concentration in serum (*P* < 0.01). The representative image of the occludin stained using immunofluorescence in the small intestinal epithelium is shown in Fig. 1. In CON group, occludin staining was diffused with less staining in the epithelium membrane, indicating possible disruption of the tight junction. Meanwhile, occludin was localized to the cell membrane region in the duodenal epithelium of piglets fed 0.2% or 1.0% TA diet. The expressions of occludin in the duodenum, jejunum and ileum were remarkably increased by 0.2% and 1.0% TA diet.

**Table 5:** Effects of tannic acid (TA) on serum parameters in weaned piglets

Item	Added tannic acid, %			SEM	*P*-value		
	0	0.2	1.0		ANOVA	Linear	Quadratic
DAO, IU/L	220.9^a^	201.3^b^	193.3^b^	3.65	< 0.001	< 0.001	0.009
*D*-Lactate, μg/mL	1.46^a^	1.36^b^	1.31^b^	0.03	0.006	0.004	0.074

CON, piglets receiving a basal diet; CON + 0.2% TA, piglets receiving a basal diet supplemented with 0.2% TA; CON + 1.0% TA, piglets receiving a basal diet supplemented with 1.0% TA; DAO, diamine oxidase

^a,b^Mean values with unlike superscript letters were significantly different (*P* < 0.05)

### Intestinal morphology

The intestinal morphology is given in Table 6. In the duodenum, dietary TA supplementation had no effect on villus height and the thickness of mucosa and duodenal wall (*P* > 0.05). Notably, the 0.2% TA group significantly reduced crypt depth and increased villus height/crypt depth ratio compared to the CON group and the 1% TA treatment (*P* < 0.05). In the jejunum, dietary TA supplementation had no effect on jejunal morphology, including villus height, crypt depth, mucosal and intestinal wall thickness (*P* > 0.05). In the ileum, no significant effect of dietary TA supplementation was observed on the thickness of mucosa and ileal wall (*P* > 0.05). Strangely, although the 1.0% TA group tended to reduce the crypt depth (*P* = 0.063), it significantly reduced the villus height compared with the CON group (*P* < 0.05).

**Table 6:** Effects of tannic acid (TA) on intestinal morphology in weaned piglets

Item	Added tannic acid, %			SEM	*P*-value		
	0	0.2	1.0		ANOVA	Linear	Quadratic
Duodenum							
Villus height, μm	354.4	337.6	357.2	14.25	0.585	0.642	0.362
Crypt depth, μm	182.4^a^	148.0^b^	175.6^a^	8.08	0.021	0.654	0.007
Villus height: Crypt depth	1.96^b^	2.30^a^	2.04^b^	0.08	0.030	0.767	0.010
Mucosal thickness, μm	794.6	747.7	847.9	40.96	0.255	0.197	0.296
Intestinal wall thickness, mm	1.40	1.38	1.56	0.06	0.124	0.053	0.545
Jejunum							
Villus height, μm	357.8	386.4	399.9	15.09	0.167	0.105	0.319
Crypt depth, μm	161.3	163.9	169.7	9.88	0.827	0.548	0.945
Villus height: Crypt depth	2.24	2.38	2.39	0.13	0.674	0.536	0.533
Mucosal thickness, μm	683.9	657.2	646.2	31.77	0.695	0.479	0.648
Intestinal wall thickness, mm	1.03	1.00	1.04	0.04	0.738	0.667	0.523
Ileum							
Villus height, μm	353.6^a^	385.3^a^	288.9^b^	21.11	0.017	0.012	0.124
Crypt depth, μm	176.7	166.1	140.9	10.05	0.063	0.021	0.794
Villus height: Crypt depth	2.01	2.38	2.04	0.15	0.195	0.626	0.085
Mucosal thickness, μm	542.0	533.2	593.0	63.85	0.777	0.508	0.821
Intestinal wall thickness, mm	1.05	1.15	1.13	0.08	0.674	0.625	0.465

CON, piglets receiving a basal diet; CON + 0.2% TA, piglets receiving a basal diet supplemented with 0.2% TA; CON + 1.0% TA, piglets receiving a basal diet supplemented with 1.0% TA

^a,b^Mean values with unlike superscript letters were significantly different (*P* < 0.05)

### MDA content in serum and small intestine

Table 7 presents the MDA content in serum and small intestine of weaned pigs. Dietary TA supplementation had no effect on MDA content in serum, duodenum and jejunum (*P* > 0.05), but 1.0% TA group significantly reduced the MDA content in the ileum compared with the CON group (*P* < 0.05).

**Table 7:** Effects of tannic acid (TA) on MDA content in serum and small intestine of weaned piglets

Item	Added tannic acid, %			SEM	*P*-value		
	0	0.2	1.0		ANOVA	Linear	Quadratic
Serum MDA, nmol/mL	3.80	3.52	3.62	0.22	0.674	0.761	0.412
Duodenal MDA, nmol/mg prot.	0.55	0.55	0.55	0.05	0.999	0.959	0.996
Jejunal MDA, nmol/mg prot.	0.82	0.55	0.76	0.12	0.265	0.858	0.111
Ileal MDA, nmol/mg prot.	1.30^a^	1.00^ab^	0.81^b^	0.11	0.023	0.014	0.179

CON, piglets receiving a basal diet; CON + 0.2% TA, piglets receiving a basal diet supplemented with 0.2% TA; CON + 1.0% TA, piglets receiving a basal diet supplemented with 1.0% TA; MDA, malondialdehyde

^a,b^Mean values with unlike superscript letters were significantly different (*P* < 0.05)

### Gene expression of tight junction protein

**Fig. 2:**
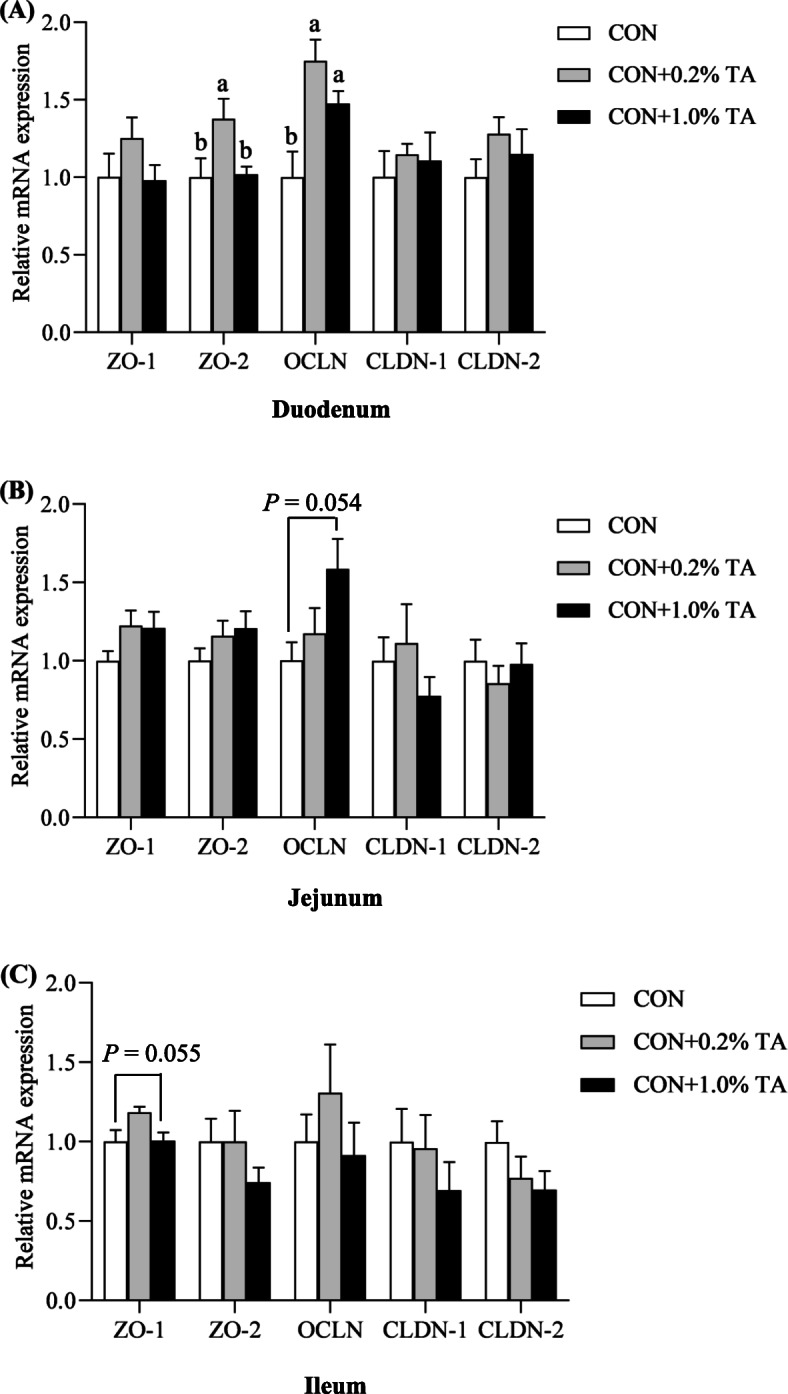
Effect of tannic acid (TA) on mRNA levels of tight junction protein-related genes in small intestine of weaned piglets. The mRNA expressions of tight junction protein-related genes in duodenum (**a**), jejunum (**b**), and ileum (**c**) of weaned piglets. CON, piglets receiving a basal diet; CON + 0.2% TA, piglets receiving a basal diet supplemented with 0.2% TA; CON + 1.0% TA, piglets receiving a basal diet supplemented with 1.0% TA; *ZO-1*, zonula occludens 1; *ZO-2*, zonula occludens 2; *OCLN*, occludin; *CLDN-1*, claudin 1; *CLDN-2*, claudin 2. The values shown represent the means ± SEM, *n* = 6; ^a,b^Mean values with unlike superscript letters were significantly different (*P* < 0.05)

The expression levels of *ZO-1*, *ZO-2*, *OCLN*, *CLDN-1* and *CLDN-2* mRNA in the small intestinal mucosa are shown in Fig. 2. In the duodenum, compared with the CON group, the higher *ZO-2* mRNA expression levels of supplemented with 0.2% TA and *OCLN* mRNA levels of supplemented with 0.2% and 1.0% TA were determined (*P* < 0.05). In addition, supplementation with 1.0% TA tended to up-regulate *OCLN* mRNA expression levels in the jejunum (*P* = 0.054), and meanwhile, similar trend was shown for *ZO-1* mRNA levels of dietary 0.2% TA supplementation in the ileum compared with the CON group (*P* = 0.055).

## Discussion

Generally, TA is unfavorable to animal growth in non-ruminant animals [20–22], but the experiment results are varied. Dietary TA supplementation at 125, 250, 500 and 1000 mg/kg levels have shown to linearly reduce ADG and feed efficiency in weanling pigs [6]. Studies in broilers showed that adding 0.07% and 0.2% TA (sweet chestnut wood extract) to the diet had no effect on ADFI, ADG and feed conversion ratio [23]. In agreement with this, the present work demonstrated that adding 0.2% or 1.0% TA to the diet had no significant effect on ADFI, ADG and F:G in weaned piglets. Moreover, dietary supplemented with 0.5% or 1% TA indicated no adverse effect on growth performance, but the higher dosage, 1.5% TA, decreased ADFI in pigs [24], suggesting the biological effects of TA are dose-dependent. On the contrary, other studies have shown that tannins could improve the growth performance of pigs and broiler chicks [13, 25, 26], or improve feed utilization and final body weight of broilers [7]. It’s well known that TA has positive effects on gastrointestinal tract of animals, such as anti-oxidation and antibacteria, however toxic and anti-nutritional effects also exist [27]. These effects seem to be closely related to the source, concentration and chemical structure of TA and animal species [5]. Thus, we hypothesized that TA concentration in our experimental diet might be not high enough to impair growth performance of weaned piglets. On the other hand, dietary tannalbin was applied to introduce TA to the pigs in our experiment. Tannalbin has a less adverse effect on the palatability of the diet, because the protein neutralizes the astringency of TA in oral cavity, which may also explain why TA has no obvious effect on the growth performance, especially the feed intake.

For a long time, post-weaning diarrhea has been treated or prevented by feed antibiotics and zinc oxide in the piglet’s feed or water [28, 29]. The development of feed strategies to promote gut health and to minimize the use of antibiotics and zinc oxide in piglets is essential for the sustainability of the pig industry. Previous studies have reported that TA is the active constituent of many medicinal plants used for treating diarrhea in rats [30]. TA has been reported as a potential treatment for diarrhea in rats [31]. Moreover, *Galla Chinensis* extract (rich in TA) also played an anti-diarrhea role in castor oil and heat-labile enterotoxin (LT)-induced diarrhea models in mice [9, 32]. Our experimental results showed that dietary supplementation of 0.2% or 1.0% TA significantly reduced the diarrhea rate of weaned piglets, which is consistent with previous research results. These indicate that TA may be a good substitute for antibiotics, zinc oxide and high copper to reduce piglet diarrhea in the era of banning antibiotics.

To understand the underlying mechanism by which TA alleviates post-weaning diarrhea, the effect of TA on intestinal barrier integrity and function has been determined. The integrity of intestinal barrier is the basis for preventing pathogenic bacteria, toxins, antigens and other harmful substances in intestinal lumen from entering blood circulation or other organs and tissues through intestinal mucosa and maintaining the stability of internal environment in animals’ body. Impaired intestinal barrier is usually a major cause of diarrhea in piglets after weaning. Therefore, improving the intestinal barrier damage caused by weaning stress is very important for relieving diarrhea in piglets. Stress related to weaning in piglets leads to impairment of the intestinal mucosal barrier with thinning of intestinal wall and increase of intestinal permeability [1, 33]. Intestinal permeability can be evaluated by some blood indexes such as DAO activity and *D*-lactic acid concentration, which are considered as quantitative and sensitive circulating markers for monitoring the degree of intestinal barrier damage [3]. DAO is a highly active intracellular enzyme existing in mammalian intestinal villi cells, and it will be released into the blood when intestinal epithelial cells and barriers are damaged [34]. Similarly, *D*-lactic acid is a bacterial metabolite existing in the intestinal lumen and permeates into the blood when the intestinal barrier is damaged [35]. The present study showed that dietary 0.2% and 1.0% TA supplementation reduced DAO activity and *D*-lactic acid concentration in serum of weaned piglets, indicating that TA is beneficial to reduce intestinal permeability.

Intestinal morphology is the most common and direct method to evaluate the integrity and function of intestinal barrier. The integrity of intestinal morphological structures plays a crucial role in maintaining normal intestinal function [36]. Acute or persistent structural and functional changes of small intestine induced by weaning stress are mainly manifested in atrophy of intestinal villi and increase of crypt depth after weaning of piglets. Such changes are known to reduce digestive and absorptive capacities and contribute to post-weaning diarrhea [37]. Diets supplemented with hydrolysable tannin increased villus height, villus perimeter and mucosal thickness of duodenum in fattening boars [12] and reduced crypt depth of ileum in weaned piglets [13]. Our finding showed that dietary supplementation of 0.2% TA significantly reduced crypt depth and increased the ratio of villus height to crypt depth in duodenum, suggesting that TA is helpful to promote the reconstruction of intestinal morphology after injury. The decrease of crypt depth may be beneficial to reduce post-weaning diarrhea of piglets because the crypts in the small intestine mainly have a secretory function [13]. Surprisingly, although the addition of 1.0% TA to the diet tended to reduce the crypt depth of ileum, it also significantly reduced the villus height. The specific reasons for this phenomenon need to be further explored.

As we know, weaning stress of piglet leads to excessive production of reactive oxygen species (ROS), which breaks the balance between oxidation system and antioxidant system, forms oxidative stress and leads to tissue damage, including intestinal barrier injury. Therefore, oxidative stress inhibition may be an effective strategy to repair intestinal barrier damage [38]. The MDA, a lipid peroxidation product, is a marker of oxidative stress and is considered as an indicator to reflect the degree of cell damage and lipid peroxidation [3, 38]. In our study, dietary supplementation of 1.0% TA significantly reduced MDA content in the ileum, which was in accordance with previous study in rats [39], indicating that TA is beneficial to reduce oxidative stress.

Tight junctions (TJs) are known to be the most important connection mode between intestinal mucosal cells, which is mainly composed of transmembrane proteins (e.g., occludin, claudins) and cytosolic proteins (e.g., ZOs), and plays a crucial role in maintaining intestinal mucosal permeability and the integrity of the epithelial barrier [40]. Weaning stress generally increases intestinal permeability via destroying TJs, such as decreasing the expression of *OCLN* and *ZO-1* for piglets [35], and leads to increased penetration of pathogenic bacteria, toxins and antigens, and increases the incidence of diarrhea [41]. In this study, addition of 0.2% or 1.0% TA to the diet significantly up-regulated the expression of *OCLN* and *ZO-2* mRNA in the duodenum, meanwhile tended to up-regulate the expression of *OCLN* mRNA in the jejunum and *ZO-1* mRNA in the ileum. Moreover, we also found that the transmembrane tight junction protein expressions were significantly increased and the localization to the cell membrane of the intestinal epithelium were improved with the addition of dietary TA by immunofluorescence analysis. The above findings suggest that TA decreased epithelial permeability, improved intestinal barrier integrity, and may help to prevent invasions of pathogens and reduce the incidence of diarrhea in pigs.

## Conclusion

In conclusion, this study has provided evidence that adding TA at a concentration of 0.2% and 1.0% to the diet can alleviate post-weaning diarrhea in piglets without reducing growth performance. Moreover, dietary TA supplementation reduced intestinal permeability, alleviated intestinal mucosal damage, and up-regulated the expression of intestinal epithelial tight junction protein, indicating that TA exerted anti-diarrhea effect on weaned piglets by improving intestinal barrier integrity and function.

## Data Availability

The datasets used and/or analysed during the current study are available from the corresponding author on reasonable request.

## Abbreviations

ADFI: Average daily feed intake
ADG: Average daily gain;
ANF: Anti-nutritional factor
CLDN-1: Claudin-1
CLDN-2: Claudin-2
CON: Control
DAO: Diamine oxidase
DAPI: 4′,6-diamidino-2-phenylindole
EDTA: Ethylene diamine tetraacetic acid
F:G: Feed to gain ratio
MDA: Malondialdehyde
OCLN: Occludin
TA: Tannic acid
ZO-1: Zonula occludens-1
ZO-2: Zonula occludens-2

## References

[1] Smith F, Clark JE, Overman BL, Tozel CC, Huang JH, Rivier JE (2009). Early weaning stress impairs development of mucosal barrier function in the porcine intestine. Am J Physiol Gastrointest Liver Physiol.

[2] Blikslager AT, Moeser AJ, Gookin JL, Jones SL, Odle J (2007). Restoration of barrier function in injured intestinal mucosa. Physiol Rev.

[3] Chen J, Yu B, Chen D, Huang Z, Mao X, Zheng P (2018). Chlorogenic acid improves intestinal barrier functions by suppressing mucosa inflammation and improving antioxidant capacity in weaned pigs. J Nutr Biochem.

[4] Ye M-H, Nan Y-L, Ding M-M, Hu J-B, Liu Q, Wei W-H, et al. Effects of dietary tannic acid on the growth, hepatic gene expression, and antioxidant enzyme activity in Brandt's voles (*Microtus brandti*). Comp Biochem Physiol B Biochem Mol Biol. 2016;196:19–26. 10.1016/j.cbpb.2016.01.011 26850644

[5] Mueller-Harvey I (2006). Unravelling the conundrum of tannins in animal nutrition and health. J Sci Food Agric.

[6] Lee S, Shinde P, Choi J, Kwon I, Lee J, Pak S (2010). Effects of tannic acid supplementation on growth performance, blood hematology, iron status and faecal microflora in weanling pigs. Livest Sci.

[7] Starčević K, Krstulović L, Brozić D, Maurić M, Stojević Z, Mikulec Ž (2015). Production performance, meat composition and oxidative susceptibility in broiler chicken fed with different phenolic compounds. J Sci Food Agric.

[8] Cappai MG, Wolf P, Pinna W, Kamphues J. Pigs use endogenous proline to cope with acorn (*Quercus pubescens* Willd.) combined diets high in hydrolysable tannins. Livest Sci. 2013;155(2–3):316–22.

[9] Yang Y, Luo H, Song X, Yu L, Xie J, Yang J, et al. Preparation of* Galla Chinensis* oral solution as well as its stability, safety, and antidiarrheal activity evaluation. Evid Based Complement Alternat Med. 2017;2017:1851459. 10.1155/2017/1851459 PMC5547719 28811824

[10] Girard M, Thanner S, Pradervand N, Hu D, Ollagnier C, Bee G (2018). Hydrolysable chestnut tannins for reduction of postweaning diarrhea: efficacy on an experimental ETEC F4 model. PLoS One.

[11] Palombo EA (2006). Phytochemicals from traditional medicinal plants used in the treatment of diarrhoea: modes of action and effects on intestinal function. Phytother Res.

[12] Bilić-Šobot D, Kubale V, Škrlep M, Čandek-Potokar M, Škorjanc D (2016). Effect of hydrolysable tannins on intestinal morphology, proliferation and apoptosis in entire male pigs. Arch Anim Nutr.

[13] Biagi G, Cipollini I, Paulicks BR, Roth FX (2010). Effect of tannins on growth performance and intestinal ecosystem in weaned piglets. Arch Anim Nutr.

[14] Montagne L, Cavaney FS, Hampson DJ, Lalles JP, Pluske JR (2004). Effect of diet composition on postweaning colibacillosis in piglets. J Anim Sci.

[15] Liu P, Piao XS, Kim SW, Wang L, Shen YB, Lee HS (2008). Effects of chito-oligosaccharide supplementation on the growth performance, nutrient digestibility, intestinal morphology, and fecal shedding of Escherichia coli and lactobacillus in weaning pigs. J Anim Sci.

[16] Huang C, Song P, Fan P, Hou C, Thacker P, Ma X (2015). Dietary sodium butyrate decreases Postweaning diarrhea by modulating intestinal permeability and changing the bacterial communities in weaned piglets. J Nutr.

[17] He X, Yu B, He J, Huang Z, Chen D. Effects of xylanase on growth performance, nutrients digestibility and intestinal health in weaned piglets. Livest Sci. 2020;233:103940.

[18] Chen J, Li Y, Yu B, Chen D, Mao X, Zheng P (2018). Dietary chlorogenic acid improves growth performance of weaned pigs through maintaining antioxidant capacity and intestinal digestion and absorption function. J Anim Sci.

[19] Livak K, Schmittgen T (2000). Analysis of relative gene expression data using real-time quantitative PCR and the 2^-△△Ct^ method. Methods..

[20] Marzo F, Tosar A, Santidrian S (1990). Effect of tannic acid on the immune response of growing chickens. J Anim Sci.

[21] Ebrahim R, Liang JB, Jahromi MF, Shokryazdan P, Ebrahimi M, Li Chen W (2015). Effects of tannic acid on performance and fatty acid composition of breast muscle in broiler chickens under heat stress. Ital J Anim Sci.

[22] Bee G, Silacci P, Ampuero-Kragten S, Čandek-Potokar M, Mueller-Harvey I (2016). Hydrolysable tannin-based diet rich in gallotannins has a minimal impact on pig performance but significantly reduces salivary and bulbourethral gland size. Animal..

[23] Rezar V, Salobir J. Effects of tannin-rich sweet chestnut (*Castanea sativa* mill.) wood extract supplementation on nutrient utilisation and excreta dry matter content in broiler chickens. Eur Poult Sci. 2014;78(1):1–10.

[24] Wang M, Huang H, Hu Y, Huang J, Yang H, Wang L (2020). Effects of dietary microencapsulated tannic acid supplementation on the growth performance, intestinal morphology, and intestinal microbiota in weaning piglets. J Anim Sci.

[25] Schiavone A, Guo K, Tassone S, Gasco L, Hernandez E, Denti R (2008). Effects of a natural extract of chestnut wood on digestibility, performance traits, and nitrogen balance of broiler chicks. Poult Sci.

[26] Brus M, Dolinšek J, Cencič A, Škorjanc D. Effect of chestnut (*Castanea sativa* mill.) wood tannins and organic acids on growth performance and faecal microbiota of pigs from 23 to 127 days of age. Bulg J Agric Sci. 2013;19(4):841–7.

[27] Inserra L, Luciano G, Bella M, Scerra M, Cilione C, Basile P (2015). Effect of including carob pulp in the diet of fattening pigs on the fatty acid composition and oxidative stability of pork. Meat Sci.

[28] Pluske JR (2013). Feed-and feed additives-related aspects of gut health and development in weanling pigs. J Anim Sci Biotechnol.

[29] Lekagul A. Patterns of antibiotic use in global pig production: a systematic review. Vet Anim Sci. 2019;7:100058. 10.1016/j.vas.2019.100058 PMC7386699 32734079

[30] Kota BP, Teoh AW, Roufogalis BD (2012). Pharmacology of traditional herbal medicines and their active principles used in the treatment of peptic ulcer, diarrhoea and inflammatory bowel disease. New Adv Basic Clin Gastroenterol.

[31] Wongsamitkul N, Sirianant L, Muanprasat C, Chatsudthipong V (2010). A plant-derived hydrolysable tannin inhibits CFTR chloride channel: a potential treatment of diarrhea. Pharm Res.

[32] Chen J-C, Ho T-Y, Chang Y-S, Wu S-L, Hsiang C-Y. Anti-diarrheal effect of *Galla Chinensis* on the *Escherichia coli* heat-labile enterotoxin and ganglioside interaction. J Ethnopharmacol. 2006;103(3):385–91. 10.1016/j.jep.2005.08.036 16213682

[33] Hu C, Gu L, Luan Z, Song J, Zhu K (2012). Effects of montmorillonite–zinc oxide hybrid on performance, diarrhea, intestinal permeability and morphology of weanling pigs. Anim Feed Sci Technol.

[34] Xiao L, Cui T, Liu S, Chen B, Wang Y, Yang T (2019). Vitamin a supplementation improves the intestinal mucosal barrier and facilitates the expression of tight junction proteins in rats with diarrhea. Nutrition..

[35] Zhao Y, Qin G, Sun Z, Che D, Bao N, Zhang X (2011). Effects of soybean agglutinin on intestinal barrier permeability and tight junction protein expression in weaned piglets. Int J Mol Sci.

[36] Gao Y, Han F, Huang X, Rong Y, Yi H, Wang Y. Changes in gut microbial populations, intestinal morphology, expression of tight junction proteins, and cytokine production between two pig breeds after challenge with *Escherichia coli *K88: a comparative study. J Anim Sci. 2013;91(12):5614–25. 10.2527/jas.2013-6528 24126267

[37] Shin T, Yi Y, Kim J, Pluske J, Cho H, Wickramasuriya S (2017). Reducing the dietary omega-6 to omega-3 polyunsaturated fatty acid ratio attenuated inflammatory indices and sustained epithelial tight junction integrity in weaner pigs housed in a poor sanitation condition. Anim Feed Sci Technol.

[38] Zhuang S, Zhong J, Bian Y, Fan Y, Chen Q, Liu P (2019). Rhein ameliorates lipopolysaccharide-induced intestinal barrier injury via modulation of Nrf2 and MAPKs. Life Sci.

[39] Zhang J, Cui L, Han X, Zhang Y, Zhang X, Chu X (2017). Protective effects of tannic acid on acute doxorubicin-induced cardiotoxicity: involvement of suppression in oxidative stress, inflammation, and apoptosis. Biomed Pharmacother.

[40] He L, Zhou X, Huang N, Li H, Cui Z, Tian J (2017). Administration of alpha-ketoglutarate improves epithelial restitution under stress injury in early-weaning piglets. Oncotarget..

[41] Ma X, Shang Q, Wang Q, Hu J, Piao X (2019). Comparative effects of enzymolytic soybean meal and antibiotics in diets on growth performance, antioxidant capacity, immunity, and intestinal barrier function in weaned pigs. Anim Feed Sci Technol.

